# Ferroptosis, a new form of cell death: opportunities and challenges in cancer

**DOI:** 10.1186/s13045-019-0720-y

**Published:** 2019-03-29

**Authors:** Yanhua Mou, Jun Wang, Jinchun Wu, Dan He, Chunfang Zhang, Chaojun Duan, Bin Li

**Affiliations:** 1 Department of Oncology, Xiangya Hospital, Central South University, Changsha, 410008 People’s Republic of China; 2 Institute of Medical Sciences, Key Laboratory of Cancer Proteomics of Chinese Ministry of Health, Xiangya Hospital, Central South University, Xiangya Road 87th, Changsha, 410008 Hunan People’s Republic of China; 3 Hunan Cancer Hospital, The Affiliated Tumor Hospital of Xiangya Medical College, Central South University, Changsha, 410008 People’s Republic of China; 4 Department of Thoracic Surgery, Xiangya Hospital, Central South University, Changsha, 410008 People’s Republic of China; 5 National Clinical Research Center for Geriatric Disorders, Xiangya Hospital, Central South University, Changsha, People’s Republic of China

**Keywords:** Ferroptosis, Apoptosis, Autophagy, NcRNAs, Cancers, P53

## Abstract

Ferroptosis is a novel type of cell death with distinct properties and recognizing functions involved in physical conditions or various diseases including cancers. The fast-growing studies of ferroptosis in cancer have boosted a perspective for its usage in cancer therapeutics. Here, we review the current findings of ferroptosis regulation and especially focus on the function of ncRNAs in mediating the process of cell ferroptotic death and on how ferroptosis was in relation to other regulated cell deaths. Aberrant ferroptosis in diverse cancer types and tissues were summarized, and we elaborated recent data about the novel actors of some “conventional” drugs or natural compounds as ferroptosis inducers in cancer. Finally, we deliberate future orientation for ferroptosis in cancer cells and current unsettled issues, which may forward the speed of clinical use of ferroptosis induction in cancer treatment.

## Background

Ferroptosis was first proposed by Dixon as a novel cell death in 2012 [1]. Unlike autophagy and apoptosis, ferroptosis is an iron-dependent and r*eactive oxygen species (*ROS)-reliant cell death with characteristics mainly of cytological changes, including decreased or vanished mitochondria cristae, a ruptured outer mitochondrial membrane, and a condensed mitochondrial membrane [2–6]. These cell abnormalities resulted from the loss of selective permeability of plasma membrane due to intense membrane lipid peroxidation and the occurrence of oxidative stress (Table 1) [7].

**Table 1:** The main features of ferroptosis, apoptosis, autophagy, necroptosis, and pyroptosis

Cell death	Ferroptosis	Apoptosis	Autophagy	Necroptosis	Pyroptosis
Biochemical features	Inhibition of xCT and reduced GSH, inhibition of GPX4. Iron accumulation and lipid peroxidation	Activation of caspases oligonucleosomal DNA fragmentation	Increased lysosomal activity	Drop in ATP levels; activation of RIP1, RIP3, and MLKL	Dependent on caspase-1 and proinflammatory cytokine releases
Morphological features	Small mitochondria with condensed mitochondrial membrane densities, reduction or vanishing of mitochondria crista, as well as outer mitochondrial membrane rupture	Plasma membrane blebbing; cellular and nuclear volume reduction; nuclear fragmentation	Formation of double-membraned autolysosomes	Plasma membrane rupture; organelle swelling; moderate chromatin condensation	Karyopyknosis, cell edema and membrane rupture
Key genes	GPX4, Nrf2, LSH, TFR1, xCT	Caspase, P53, Fas, Bcl-2, Bax	ATG5, ATG7, DRAM3, TFEB	LEF1, RIP1, RIP3	Caspase-1, IL-1β, IL-18
Regulatory pathways	xCT and Gpx4, MVA, HSF1-HSPB1, p62-Keap1-Nrf2 pathway, LSH signal pathway	Death receptor, Mitochondrial, Endoplasmic reticulum pathway Csapase, P53, Bcl-2 mediated signaling pathway	PI3K-AKT-mTOR,MAPK-ERK1/2-mTOR signal pathway	TNFα,TNFR1,TLR3, TRAIL, FasL, ROS, PKC-MAPK-AP-1-mediated signaling pathway	Caspase-1, NLRP3-mediated signaling pathway.
Released DAMP	HMGB1	Ecto-CRT, HMGB1, and ATP	HMGB-1	DNA and IL-6	HMGB1, ATP, IL-1β, and IL-18
Immune features	Pro-inflammatory	Mostly anti-inflammatory	Mostly anti-inflammatory	Mostly pro-inflammatory	*pro-inflammatory*
Inducers	Erastin,DPI2, BSO, SAS, lanperisone, SRS, RSL3, DPI7, DPI10, FIN56, sorafenib, artemisinin	FASL, DCC, UNC5B	Rapamycin, lithium, sodium, valproate, carbamazepine, C2-ceramide, rapamycin	TNFa, zVAD-fmk, PAMPS	ZnO—NPs, Ivermectin
Inhibitors	Desferoxamine, vitamin E, U0126, ferrostatin-1, SRS, CA-1, cycloheximide, aminooxyacetic acid Liproxstatin-1 HCl	XIAP, c-IAP1, c-IAP2, ILP-2, ML-IAP/livin, NAIP, Z-VADFMK	3-ME, LY294002, wortmannin, PIK-III, compound 31, SAR 405, Vps34-In1,MRT68921, Spautin-1, Bafilomycin A1, hydrochloroquin	Nec-1, NSA, Kongensin- A	Necrosulfonami-de

Researches indicated that ferroptosis could be triggered by diverse physiological conditions and pathological stresses in humans and animals [8]. Ferroptosis is gradually accepted as an adaptive feature to eliminate the malignant cells. It plays a pivotal role in the depression of tumorigenesis by removing the cells that are deficient in key nutrients in the environment or damaged by infection or ambient stress [9]. Studies have shown that the classic oxidative stress pathway was an important causative factor to induce ferroptosis. Although cancer cells are under continuous oxidative stress with an exquisite balance between thiols and catalytic iron, ferroptosis does not often happen in the cancer development [10]. The underlying molecular mechanisms remain poorly understood. We herein review the occurrence and regulation of ferroptosis in various cancer cells. The opportunity and challenge of cancer treatment based on ferroptosis will be detailed, which was desired to prosper new strategies for cancer therapy of clinical value.

### Ferroptosis, from a cancer perspective: an overview

Ferroptosis was first observed in oncogenic Ras-expressing human foreskin fibroblast cell line by a battery of small compounds considered as ferroptosis-*inducing agents* (FIN), including erastin and Ras-selective lethal small molecule 3 (RSL 3). With the following studies, the relationship of Ras oncoprotein with ferroptosis becomes agnostic. Some Ras WT cells including fibrosarcoma cells, kidney tubule cells, and T cells are vulnerable to erastin, but the RMS13 rhabdomyosarcoma cells with Ras mutation were resistant to erastin and RLS3. Indeed, the ferroptosis inducer artesunate/erastin can promote ferroptosis in a Ras-reliant way in pancreatic cancer or transformed fibroblastic cells, while in a Ras-independent manner in leukemia cells [6, 11].

**Fig. 1:**
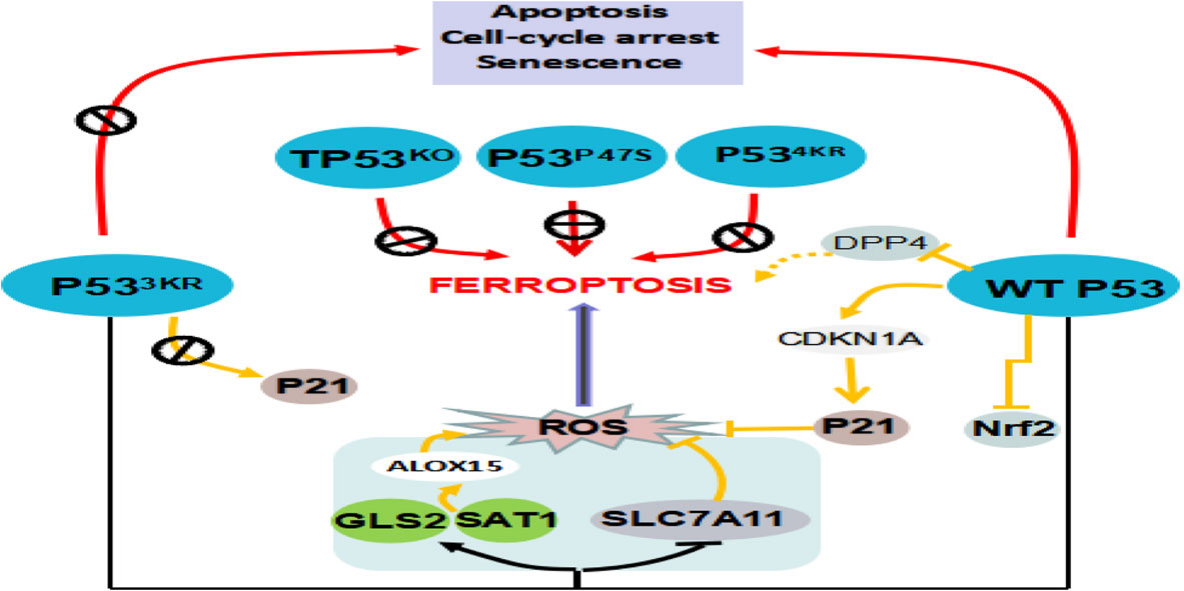
P53 regulate ferroptosis. P53^3KR^ cannot elicit apoptosis activity, it retains the ability to promote ferroptosis. GLS2 and SAT1 contribute, at least in part, to P53^3KR^-mediated ferroptosis. P53^3KR^ completely retains the ability to regulate the expression of SLC7A11. Interestingly, WTP53 inhibits ferroptosis via blocking the DPP4 activity. And WTP53 can delay ferroptosis by promoting the expression of P53 transcriptional target CDKN1A. However, P53^3KR^ is unable to induce P21. Other P53 mutants, P53^4KR^, P53^P47S^, and TP53^KO^, cannot induce ferroptosis and weaken the blocking of cell growth

Emerging evidence implicated that ferroptosis may be an adaptive process which was critical for eradicating the carcinogenic cells [1]. More clues for this role of ferroptosis can be derived from recent researches of the *tumor suppressor P53 (*TP53). The acetylation-defective mutant TP53^3KR^ lost the ability to induce cell senescence, apoptosis, and cell-cycle arrest, which were the main functions of TP53 in tumor suppression. Impressively, TP53^3KR^ can still hold the capacity of inhibiting tumorigenesis due to its ferroptosis induction [12–14]. An argument does emerge that P53 expression may promote, limit, or detain the outset of ferroptosis in certain cells or conditions (Fig. 1). These opposite jobs of p53 in operating the process of ferroptotic cell death were executed by different mechanisms, including effects on metabolic genes transcription, post-translational regulation or by virtue of P53-P21 axis [15, 16]. The bidirectional regulation of ferroptosis by P53 in a cell-specific or context-dependent manner needs to be further investigated. Moreover, it is still obscure that what kind of role P53-target genes take part in manipulating of the ferroptotic cell death [17]?

**Fig. 2:**
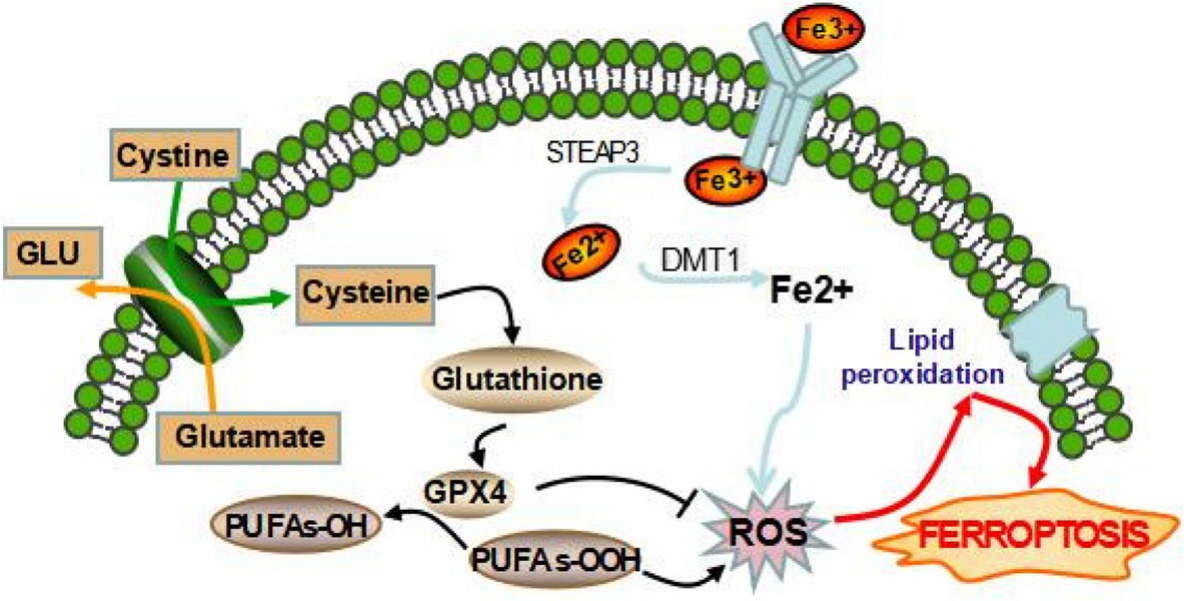
Mechanism of ferroptotic cell death. System xc- transports intracellular Glu to the extracellular space and extracellular Cys2 into the cell, which is then transformed into Cys for GSH synthesis. GPX4 reduces the endogenous neutralization of PUFAs-OOH to PUFAs-OH, ultimately reducing ROS accumulation. Excess irons are the basis for ferroptosis execution. Circulated iron was combined with transferrin in the form of Fe3+, and then it entered into cells by TFR1. Iron in Fe3+ form was deoxidized to iron in Fe2+ by iron oxide reductase STEAP3. Ultimately, Fe2+ was released into a labile iron pool in the cytoplasm from the endosome mediated by DMT1

Ferroptosis is programmed necrosis mainly triggered by extra-mitochondrial lipid peroxidation arising from an iron-dependent ROS accretion. Excessive iron originally from aberrant iron metabolism or maladjustment of two major redox systems (lipid peroxidation and thiols) was the main incentive factors of ROS production. Glutathione (GSH), a thiol-containing tripeptide, synthesis is determined by the constant import of cysteine (Cys2) by the cell surface Cys2/glutamate antiporter xCT (Fig. 2).

**Fig. 3:**
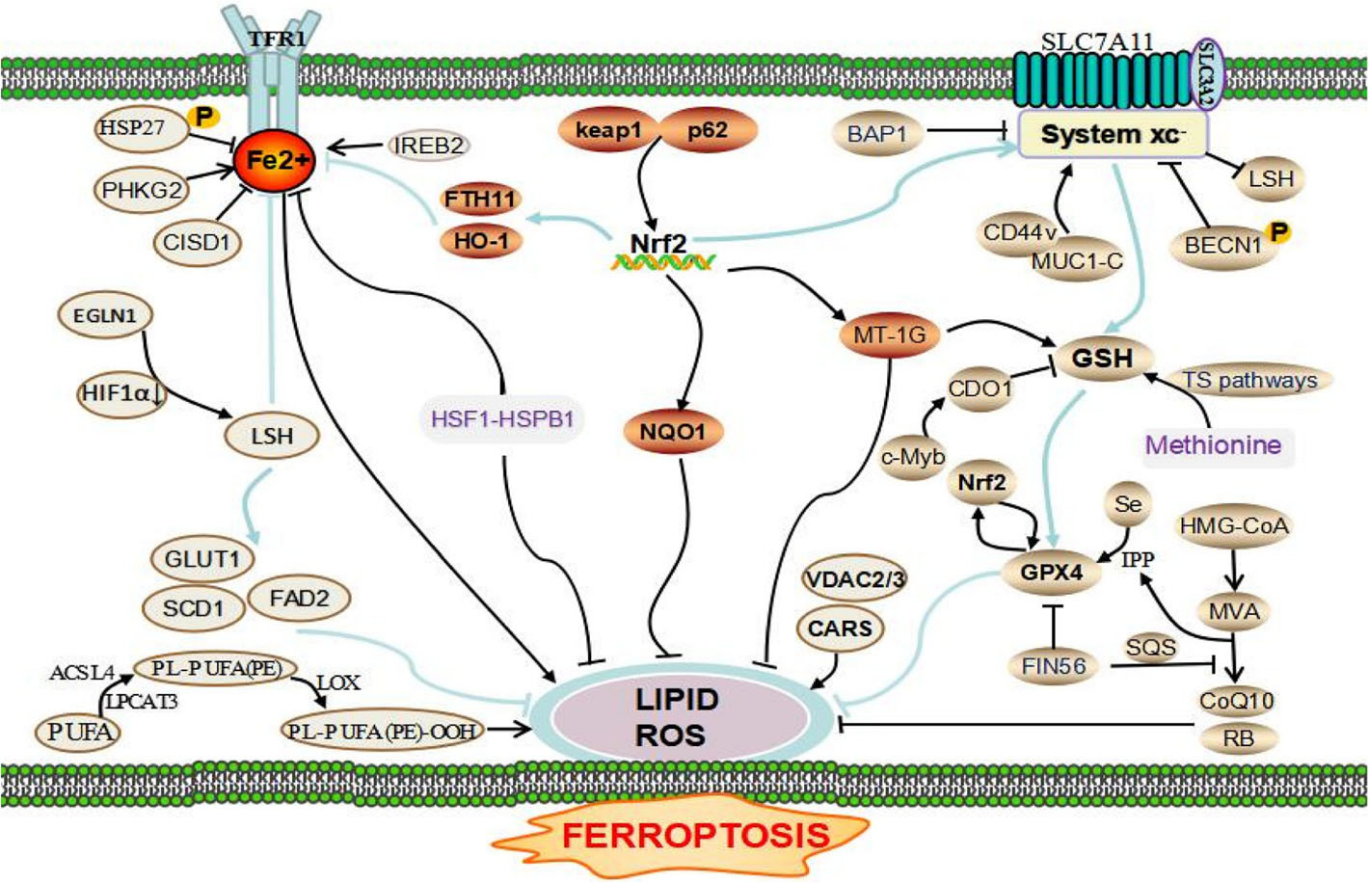
The regulatory network of ferroptosis. PHKG2, IREB2, and CISD1 play an important role in ferroptosis by their function in iron metabolism balance. The phosphorylation of HSP27 induces ferroptosis resistance through blocking cytoskeleton-mediated iron absorption. EGLN1 can upregulate LSH expression by limiting HIF 1α. LSH inhibits ferroptosis by affecting metabolism-associated genes including SCD1, GLUT1, and FADS2. Also, protein kinase C-mediated HSPB1 is a negative regulator of ferroptosis by inhibiting ROS production and reducing iron uptake. The p62-Keap1-Nrf2 pathway plays a vital role against ferroptosis by regulating Nrf2-targeted genes HO-1, FTH1, and NQO1. AMPK-mediated BECN1 phosphorylation and BAP1 directly represses system xc- activity, leading to the elevated ROS level and ferroptosis. MUC1-C binding with CD44v promotes the stability of system xc-. The inhibition of CDO1 restores the levels of GSH and increases ROS. Methionine can be converted to S-adenosylhomocysteine and Cys through the sulfur transfer pathway, which is essential for GPX4 biosynthesis. IPP and CoQ10 are the important products of the MVA pathway, which promotes GPX4 synthesis. FIN56 treatment also reduces CoQ10 by modulating SQS. VDAC2/3 and CARS are positive regulators of ferroptosis. ROS accumulation requires the activation of PUFAs by ACSL4 and LPCAT3. And LOX directly catalyzes the peroxidation of phospholipid PUFAs

The activation of Ras-mitogen-activated protein kinase (MEK) signaling can attribute to the sensitivity of cancer cells to ferroptosis, resulting from its promoting iron abundance in cancer by governing the expression levels of the transferrin receptor and ferritin [2, 7, 8]. And the overactive Ras-MEK pathway may enhance ROS generation via inhibiting cystine (Cys2) uptake or mitochondrial voltage-dependent anion channel 2/3 (VDAC 2/3) and consequently sensitize cancer cells to ferroptosis [18, 19]. But scientist and skeptics argued that the conclusion is unreliable because the MEK inhibitor U0126 was used in these above studies. Compared with U0126, the special MEK1/2 inhibitor, PD0325901 cannot halt cell ferroptotic cell death induced by FIN. So MEK activity is not indispensable for ferroptosis [20, 21]. Other signal pathways were also pinpointed to regulate the process of cell ferroptotic death, e.g., *Keleh-like ECH-associated protein 1 (*Keap1)-nuclear factor erythroid 2-related factor 2 (Nrf2), lymphoid-specific helicase (LSH), Egl nine homolog 1 (EGLN1)/cellular myelocytomatosis oncogene (c-Myc), mevalonate (MVA), sulfur-transfer, mucin 1 C-terminal (MUC1-C)/*system xc*- (xCT) and heat shock factor-1 (HSF1)-heat shock protein beta-1 (HSPB1) pathway [5]. RNAi against Fms-like tyrosine *kinase* 3 (Flt3) saves cells from ferroptosis by limiting lipid peroxidation and inactivating p22phox which back the role for Flt3 kinase in ferroptosis. A new study indicates that adenosine 5′-monophosphate-activated protein kinase (AMPK)-mediated the phosphorylation of Beclin1 (BECN1) directly blocks the activity of system xc- and thus results in the occurrence of ferroptosis [22, 23]. Recent investigations have unraveled a perplexing network in the ferroptosis regulation which was shown in Fig. 3.

Interestingly, microRNA and long non-coding RNA (lncRNA) are increasingly recognized as the crucial mediators in the regulation of ferroptosis (Table 2). The cytosolic lncRNA P53RRA can promote ferroptosis via nuclear sequestration of P53. P53RRA interplays with the *RNA recognition motif* (RRM) domain of Ras GTPase-activating protein-binding protein 1 (G3BP1), resulting in the combination of G3BP1 with P53, which cause more P53 custody in the nucleus and less sequestration of p53 in the cytoplasm [24]. P53RRA increased the intracellular concentrations of iron and lipid ROS by means of the augmented P53 pathway. Taken together, the cytosolic P53RRA-G3BP1 is a novel mechanism for inducing ferroptosis in lung adenocarcinoma.

**Table 2:** Non-coding RNA associated with ferroptosis

ncRNA	Target	Cell lines	Mechanisms of action	Function	Ref
P53RRA	p53	A549, H522, SPCA1	Leads to higher retention of p53 in the nucleus, increases lipid ROS and iron concentrations	Promote	[24]
miR-9	GOT1	A375, G-361	Suppressed GOT1, which ultimately converts Glu to a-KG	Suppress	[25]
miR-137	SLC1A5	A375, G-361	Suppressed SLC1A5, resulting in decreased Gln uptake and malondialdehyde (MDA) accumulation	Suppress	[26]
miR-375	SLC7A11	MCF7	Effectively suppress the expression of SLC7A11	Promote	[27]
miR-27a		EJ/T24, RT112			[28]
miR-26b		CAL-27, Tca8113VC			[29]
As-SLC7A11		A433, OVCA429, TOV112D			[30]
miR-7	Nrf2	SH-SY5Y	Activates Nrf2 pathway by targeting Keap1 expression	Suppress	[31]
miR-200a		MCF-10A, MDA-MB-231			[32]
miR-101		HUVECs	Activate Nrf2 signaling by directly targeting Cul3		[34]
miR-455		hFOB1.19			[35]
miR-153		SH-SY5Y	Directly target Nrf2 and downregulate expression of Nrf2	Promote	[36]
miR-142-5p					
miR-27a					
miR-144		K562, SH-SY5Y	Reduced levels of Nrf2, decreased GSH		[37]
miR-93		MCF-10A, T47D	Decreased protein expression of Nrf2 and Nrf2- regulated genes		[38]
miR-34a		NRK-52E, HK-2	Decreased levels of Sirt1, which is required for the activation of Nrf2 system		[39]
miR-28		MCF-7	Inhibits Nrf2 expression through a Keap1- independent manner		[33]
miR-365-1		BEAS-2B, A549, 3 T3-L1	Decreased protein expression of Nrf2 genes		[40]
miR-193b					
miR-29-b1					
miR-20a	FPN	Huh7, NSCLC	Represses FPN expression by directly targeting the FPN 39UTR	Promote	[41]
miR-485-3p		HepG2, K562			[42]
miR-210	TFR	MCF7	Decreases the uptake of transferrin by inhibiting the expression of TFR	Suppress	[43]
miR-152	TFR1	SK-HEP1, HepG2	Effectively inhibit the expression level of TFR1		[44]
miR-200b	FTH	MDA-MB-231	Effectively inhibit the expression levels of FTH		[45]
miR-Let-7d	DMT1-IRE	K562, HEL	Reduces iron accumulation and simultaneously regulates the expression level of DMT1-IRE		[46]
miR-3595	ACSL4	HSC-T6	Inhibits the expression of mRNA and protein in ACSL4	Suppress	[47]
miR-205		HepG2			[48]
miR-224-5P		3 T3-L1			[49]
miR-19b-3p		CaCO_2_	Suppression the expression of ACSL4		[50]
miR-130a-3p					
miR-150-5p					
miR-144-3p					
miR-16-5p					
miR-7a-5p					
miR-17-5p					
MiR-206	ROS	Adult mongrel dogs	Increased the production of ROS by targeting SOD1	Promote	[51]
miR-155		Capan-2, Aspc-1	Increases ROS levels through inhibiting Foxo3a expression		[52]
miR-25		Rats	Restraining ROS level by targeting NOX4.	Suppress	[53]
miR-448-3p		Mice	Reduced NOX2- dependent ROS production		[54]

In melanoma cell lines G-361 and A375, miR-9 can inhibit ferroptosis via targeting glutamic-oxaloacetic transaminase 1 (GOT1), an enzyme via glutaminolysis converting glutamine (Gln) ultimately to α-ketoglutarate (α-KG), which can promote ROS accumulation and thus irritate ferroptosis [25]. Knockdown miR-9 elevated the level of GOT1 and a-KG, which subsequently increased the sensitivity of cells to erastin- and RSL3-induced ferroptosis. Intriguingly, the other study showed that miR-137 impedes ferroptosis through directly suppressing s*olute* c*arrier* f*amily 1* member 5 (SLC1A5), which is a major receptor for Gln uptake [26]. Knockdown of miR-137 can enhance the antitumor activity of erastin by enhancing ferroptosis.

As a specific light-chain submission of the *Cys2*/glutamate (Glu) antiporter, solute carrier family 7 member 11 (SLC7A11) plays a critical role in the negative regulation of ferroptosis. Researches showed that miR-375, miR-27a, miR-26b, and As-SLC7A11 (antisense lncRNAs) could suppress the transcription of SLC7A11 mRNA and impair the strength of its protein [27–30]. So, it is plausible that these miRNAs can promote ferroptosis by targeting SLC7A11.

The Nrf2 is the vital inhibitor of ferroptosis due to its ability to inhibit cellular iron uptake, limiting ROS production, and upregulating SLC7A11. First, miR-7 and miR-200a readily induce the activation of the Nrf2 pathway by repressing Keap1 expression [31, 32]. Contrastingly, miR-28 suppresses Nrf2 expression in a Keap1-independent way [33]. Second, both miR-101 and miR-455 can promote Nrf2 nuclear accumulation by targeting Cullin-3 (Cul3) [34, 35]. Finally, miR-153, miR-142-5p, miR-27a [36], miR-144 [37], miR-93 [38], miR-34a [39], miR-365-1, miR-193b, and miR-29-b1 [40] can decrease Nrf2 level through different mechanisms. These results indicated that miRNA might modify ferroptosis by means of regulating the expression of Nrf2.

It was confirmed that iron overload could contribute to ferroptosis in cancer. The iron metabolism-related genes, such as transferrin (TF), transferrin receptor 1 (TFR1), ferroportin (FPN), divalent metal transporter 1 (DMT1), *ferritin heavy chain* 1 (FTH1), *and ferritin light* chain (FTL), were the critical mediators in the ferroptosis procedure. Based on the existing research findings, miRNAs were also involved in the regulation of iron export, storage, utilization, and uptake. MiR-20a and miR-485-3p can reduce iron output by targeting FPN genes [41, 42]. MiR-210 and miR-152 inhibit the expression level of TFR, thereby reducing the uptake of TF [43, 44]. Concurrently, the expressions of miR-200b and miR-Let-7d effectively reduce iron accumulation by inhibiting the expression of FTH and DMT1-iron-responsive element (IRE), respectively [45, 46].

Beyond that, previous studies have confirmed that ROS generation requires the activation of polyunsaturated fatty acids (PUFAs) by Acyl-CoA synthetase long-chain family member 4 (ACSL4) and lysophosphatidylcholine acyltransferase 3 (LPCAT3). MiR-3595 [47], miR-205 [48], miR-224-5P [49], miR-19b-3p, miR-130a-3p, miR-150-5p, miR-144-3p, miR-16-5p, miR-7a-5p, and miR-17-5p [50] can decrease the expression of ACSL4. It is conceivable but not yet demonstrated that these miRNAs can regulate ferroptosis by targeting ACSL4.

Ultimately, ROS is the indispensable molecule in the process of ferroptosis. Previous studies have confirmed that miRNAs are closely related to redox signaling and ROS production, and we can further speculate that miRNAs can regulate ferroptosis by regulating the expression of ROS. MiR-206 significantly induces ROS accumulation by binding to the mRNA of superoxide dismutase 1 (SOD1) [51]. MiR-155 increases the generation of ROS by inducing Foxo3a deficiency [52]. Also, miR-25 and miR-448-3p have been proved to reduce ROS level by targeting the nicotinamide adenine dinucleotide phosphate-*oxidase* (NOX) [53, 54].

### Ferroptosis, as an expanding network of programmed cell death in cancer

Apart from apoptosis and autophagy, other programmed cell death such as programmed necrosis was discovered. Ferroptosis, as well as necroptosis, parthanatos, and pyroptosis, are all belonging to the programmed necrosis, which was carried out by a specific program of genetically encoded cellular machinery demolishing the cell in an ordered fashion [9]. Over the latest 5 years, an astonishing boost in our perception of ferroptosis has been seen, and it was linked with apoptosis, autophagy, and other programmed necrosis which are tentatively investigated.

### Ferroptosis and apoptosis: switch, synergism, or antagonism?

Recently, a growing research suggested the interconnection of ferroptosis and apoptosis. Besides preventing tumorigenesis by cell-cycle arrest and cell apoptosis induction, the canonical tumor suppressor protein P53 can also induce ferroptosis in certain conditions. Zheng et al. designed a novel type of P53 complex named metal organic network-P53 (MON-P53), which is tannic acid integrated with ferric ions forming MON on the external of the P53 plasmid [55]. When MON-P53 was internalized, ferric ions can induce Fenton reaction which will cause ROS generation. In vivo and vitro experiment, the dominant ferroptotic cell death, apart from cell apoptosis, was observed in the MON-P53-treated cells. The tumor growth was suppressed, and the life span of tumor-bearing mice was also prolonged. Therefore, this approach will direct a ferroptosis/apoptosis hybrid anti-cancer therapy [9].

**Fig. 4:**
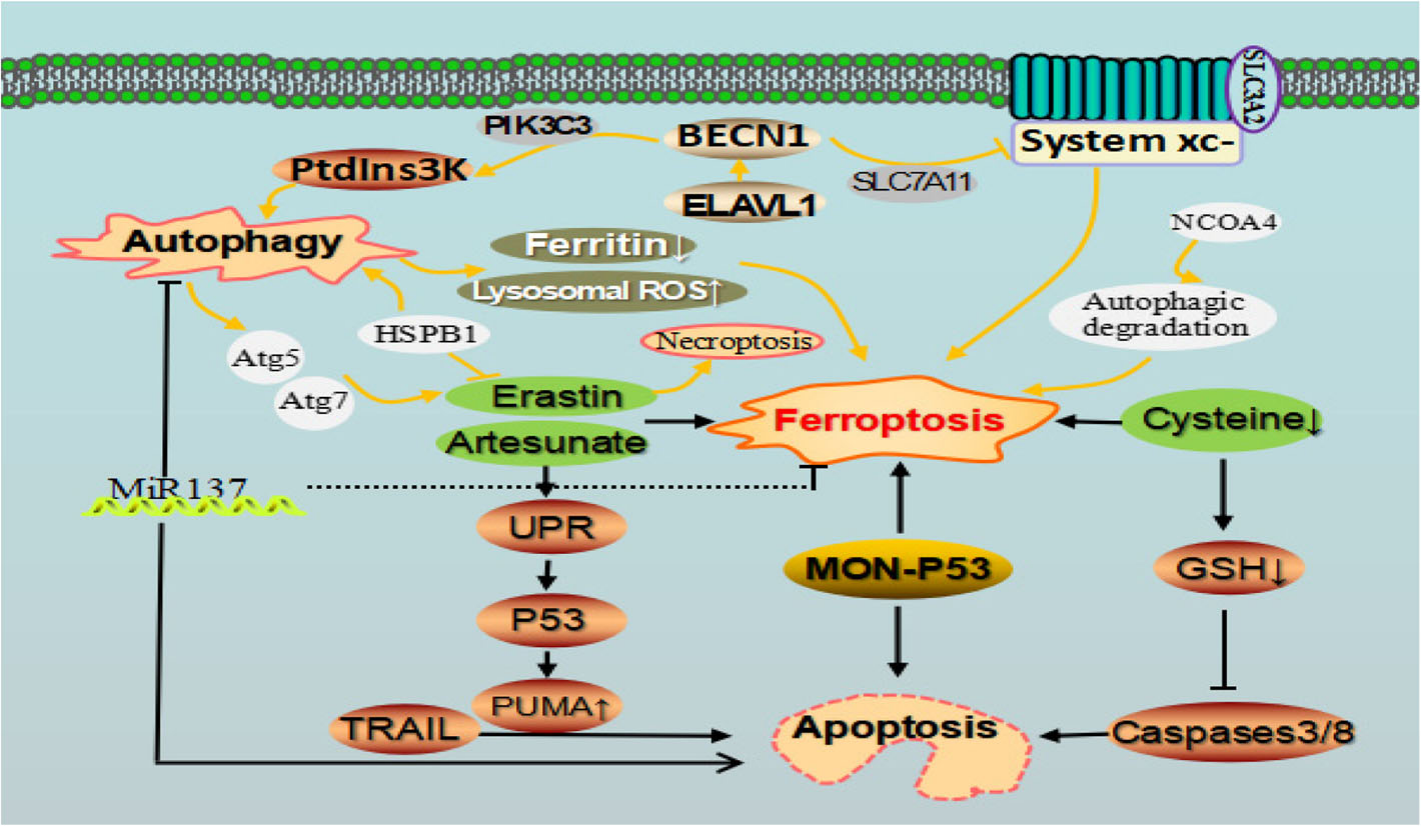
Cross-talk between ferroptosis, apoptotic, and autophagy in cancer. Autophagy can modulate cell sensitivity to ferroptosis through various pathways. Erastin, artesunate, MON-P53, and Cys closely link ferroptosis with apoptosis. ELAVL1 promoted autophagy by binding to the AU-rich elements within the F3 of the 3′-untranslated regions of BECN1 mRNA. BECN1 can promote ferroptosis via directly blocking system xc-

Conversely, ferroptotic agents such as artesunate and erastin can induce the unfolded protein response (UPR) which sequentially promotes the expression of P53 *upregulated modulator of apoptosis* (PUMA) via C/EBP-homologous protein (CHOP) in the P53-independent way [56]. PUMA can enhance the function of apoptotic agent tumor necrosis factor-related apoptosis-inducing ligand (TRAIL) in promoting cell apoptosis (Fig. 4). This observation of ferroptotic agent-mediated sensitization to TRAIL-induced apoptosis implies ferroptosis inducer combined with TRAIL can strongly augment the tumoricidal efficacy. Another opinion is that metabolic or other alterations related to the irritation of ferroptosis biochemically block apoptosis occurrence. Cells that were subjected to ferroptosis due to cysteine (Cys) deprivation have about 10% of the normal level of intracellular glutathione (GSH). The power of reduced GSH may be necessary for the cascade activation of caspases-3 and-8. Therefore, cells’ lack of GSH cannot activate caspases correctly [10, 57].

### Autophagy degrades ferritin to facilitate ferroptosis

Autophagy is a lysosome-dependent degradation pathway. The study provided data that the activation of autophagy pathway can degrade ferritin and then trigger ferroptosis in cancer cells [58]. Ferritinophagy, the autophagic turnover of ferritin, is critical to induce ferroptosis [59]. Further study indicated that BECN1 generation from the stimulation of upregulated embryonic lethal, abnormal vision, Drosophila-like 1 (ELAVL1) was responsible for the activation of autophagy in erastin- or sorafenib-irritated ferroptosis [60].

Autophagy can contribute to ferroptosis via the generation of lysosomal ROS and providing available labile iron via NCOA4-mediated ferritinophagy. And pharmacological blockage of autophagy weakens drug-induced ferroptosis in cancer cells. Genetic knockout of autophagy-related 5 (Atg5) and Atg7 limited erastin-induced ferroptosis by decreasing lipid peroxidation and intracellular ferrous iron levels. Significantly, blockage of nuclear receptor coactivator 4 (NCOA4), which was a choosy cargo receptor for ferritinophgay, suppresses ferritin degradation and inhibits ferroptosis. Contrarily, NCOA4 overexpression reinforces ferritin degradation and then drives ferroptosis. Autophagy supplies available labile iron via NCOA4-mediated ferritinophagy to the process of ferroptosis [61–63]. These results unraveled the molecular interactions between ferroptosis and apoptosis, and ferroptosis is an autophagic cell death process [64]. But in the absence of ferroptosis, prolonged iron mediates ROS accumulation and triggers autophagy and then results in autosis. Ferroptosis and autophagy can motivate cell death occurrence separately in breast cancer cells with siramesine and lapatinib treatment [58]. But contrastly, Buccarelli et al. reported that the blockage of autophagy using quinacrine can enhance glioblastoma stem cells (GSC) sensitivity to temozolomide by means of ferroptotic cell death [65].

Recent research shows that SOCS1 is required for p53 activation and the regulation of cellular senescence. SOCS1 can regulate the expression of p53 target genes such as reducing the expression of the cystine transporter SLC7A11 and the levels of glutathione, and it therefore sensitize cells to ferroptosis [66].

Although the mechanism among diverse types of regulated cell death has distinctive morphological and biochemical traits, some crosstalk still prevails between regulators and components of these various processes [67, 68]. The pathway of ferroptosis was linked with that of oxytosis by transactivating BH3-interacting domain death agonist (BID), the *pro-apoptotic* member of *Bcl-2 family proteins*, which converged to mitochondrial damage [69]. MiR137 was identified as a central mediator among apoptosis, autophagy, and ferroptosis [26]. HSPB1 is a small heat-shock protein that is crucial in controlling autophagy and ferroptosis [70]. Erastin can simultaneously induce the ferroptosis and necroptosis in HL-60 cell line [6]. Therefore, explicating how these pathways of regulated cell death are interplayed at the molecular level and how these pathways could be mapped and integrated will advance new ways to systematical research on this field. Discerning the critical factors such as ncRNAs should enable these processes to be therapeutically targeted and would be highly desired.

### Aberrant ferroptosis in diverse cancer types and tissues

The susceptibility of different types of cancer cells to ferroptosis was significantly different. NCI-60, a panel of different cancer cell lines from eight various tissue types, recommended by the US National Cancer Institute Developmental Therapeutics Program. Among them, diffuse large B cell lymphomas and Renal cell carcinoma are more susceptible to erastin-induced ferroptosis than other cancer cells from the six tissues (the breast, lung, colon, melanocytes, central nervous system, and ovary) [6]. Some argue that the sensitivity of different cell lines to ferroptosis is different because of the difference of their basic metabolic state. Numerous studies have confirmed the pivotal role of ferroptosis in killing cancer cells and suppressing cancer growth. Further investigations showed that chemotherapeutic drugs such as cytarabine/ara-C, cisplatin, doxorubicin/Adriamycin, and temozolomide combining with the ferroptosis inducer erastin gained a remarkable synergistic effect on their anti-tumor activity [3]. The prognosis is better than traditional chemotherapy alone. Here, we summarize the possible mechanism of ferroptosis in various cancer types and putative indictor of ferroptosis for clinical application.

#### Hepatocellular carcinoma

Ferroptosis was one of the underlying mechanisms in sorafenib treating HCC. HCC cells with the *retinoblastoma* (RB) protein deficiency had 2–3 times higher death rate more than that of cells with a normal level of RB protein [71]. This susceptibility of HCC with deactivated RB protein to ferroptosis was due to the augment of oxidative stress response in cells from increased reactive oxygen concentration in mitochondria. Metallothionein-1g (MT-1G) is a novel negative regulator of ferroptosis in HCC. MT-1G knockdown contributed to sorafenib-induced ferroptosis by increasing lipid peroxidation and GSH depletion. CDGSH iron sulfur domain 1 (CISD1) and ACSL4 inhibition promote erastin-induced ferroptosis in HCC. Low-density lipoprotein (LDL)–docosahexaenoic acid (DHA) nanoparticles cause cell death in HCC cells through the ferroptosis pathway. The p62-Keap1-Nrf2 pathway plays a vital role in saving HCC cells from ferroptosis, and Ras/Raf/MEK pathway is reported to be a critically important target for ferroptosis in treating HCC [72].

#### Colorectal cancer

Xie et al. reported that colorectal cancer was resistant to ferroptosis resulting from the inhibition of dipeptidyl-peptidase-4 (DDP4) activity by TP53 in a transcription-independent way [12]. P53 loss promotes DDP4 gathering to plasma-membrane and thus augments DDP4-dependent lipid peroxidation, eventually causing ferroptotic cell death.

#### Gastric cancer

Hao et al. found that erastin irritates ferroptosis in GC cells [73]. Ferrostatin-1 and liproxstatin-1 can reverse this effect. C-Myc increases the expression of cysteine dioxygenase type 1 (CDO1), facilitating ferroptosis occurrence. Mechanistically, CDO1 suppression leads to the resistance of GC cells to erastin-induced ferroptosis by restoring cellular glutathione peroxidase 4 (GPX4) expression and GSH levels, and also by decreasing ROS generation. Another study reported that the CD44 variant (CD44v) stabilizes xCT at the plasma membrane and increases Cys2 uptake for GSH synthesis, blocking the ROS-induced stress signaling, and thus confer to ferroptosis resistance in GC cell [74, 75].

#### Ovarian cancer

Ovarian cancer cells are characteristics of ferroptosis susceptibility because of excess iron overload by its tumor-initiating cells (TICs), which have overexpressed TFR1 and decreased iron efflux pump FPN level [76]. Artesunate (ART) can induce ferroptosis in a ROS-dependent way in ovarian cancer. Ferrostatin-1 can significantly reverse ART-induced cell ferroptosis, but transferrin pretreatment augments the ferroptosis of ovarian cancer cells induced by ART via enhancing cellular iron level [77].

#### Prostate adenocarcinoma

Erastin induces ferroptosis in Ras-carrying human prostate adenocarcinoma cells. The phosphorylation of HSF1-dependent HSPB1 contributes to the ferroptosis resistance to erastin through inhibiting lipid ROS accumulation and iron uptake [70]. HSPB1 inhibition specifically increased erastin-induced ferroptosis by facilitating iron accumulation from the upregulation of TFR1 and slight reduction of FTH1 expression.

#### Breast cancer

Ma et al. reported that siramesine and lapatinib induce ferroptosis by increasing iron-dependent ROS productions, and CDO1 overexpression can exacerbate ferroptotic cell death by the further accumulation of high-level ROS result from the decreased GSH levels in breast cancer cells [58]. In contrast, MUC1-C can upregulate the GSH expression by its formation of a complex with CD44v, which cause the cripple of ferroptosis in breast cancer cells [74].

#### Lung cancer

Ferroptosis of lung cancer cell was first induced by erastin in the K-ras mutated A549 cells [2]. And the following report shows that erastin sensitizes lung cancer cells to cisplatin in ferroptosis manner by GSH reduction and GPXs inactivation [19]. Cysteine desulfurase (NFS1), as an iron-sulfur cluster biosynthetic enzyme, can protect cells from ferroptosis under the high-oxygen tension by sustaining the iron-sulfur cofactors. Coinhibition of NFS1 and Cys transport can evoke ferroptosis in vitro and suppress tumor growth [20].

#### Rhabdomyosarcoma

High level of GSH biosynthesis is essential for RMS cells to grow and become multidrug resistant. GPX4 inhibition using RSL3 and erastin can induce ferroptosis in RMS13 cells by lessening GSH level [21]. Another possible reason for high susceptibility of RMS13 cell lines to erastin and RSL3 was related to its higher activity of intrinsic Ras/e*xtracellular signal-regulated kinase* (ERK). But contrastingly, the RMS13 cells with oncogenic Ras mutation were resistant to the oxidative stress-induced ferroptosis.

#### Hematological malignancies

Diffuse large B-cell lymphoma (DLBCL) cells were notably one of the most sensitive to ferroptosis inducer in the eight cell lines harvested from various tissues [78]. It is proved that the enhanced sensitivity might be due to its weakness in the sulfur transfer pathways, which causes more extracellular Cys and Cys2 required for cells survival [79]. Yu et al. reported low-dose erastin could remarkably increase the ability of cytarabine and doxorubicin to kill non-APL acute myeloid leukemia (AML) cells by irritating both necroptosis and ferroptosis [80].

### Ferroptosis-induction cancer therapy

Since erastin, a novel compound is found in human tumor cells in 2003 [81]. It was first identified as a ferroptosis inducer in 2012 [8], several clinical drugs have also been found to hold a capacity of inducing ferroptosis in cancer cells.

## Chemotherapeutic agents

### Sulfasalazine

SSZ is recently recognized as a system xc- inhibitor [41]. xCT expression has circadian rhythm and the expression of TFR1 was affected by the circadian organization of molecular clock. Bmal1 and the clock regulate the circadian rhythm of xCT expression. The clock-controlled gene c-Myc rhythmically activated the transcription of the TfR1 gene. SSZ has been reported to disrupt the circadian rhythm of transferrin receptor 1 gene expression and thus it was plausible that SSZ may affect iron metabolism [82–84]. Toyokuni et al. report that sulfasalazine inhibits Cys2 uptake via system xc-, resulting in ferroptosis in glioma cells [85]. Based on the circadian rhythm of xCT, SSZ has different effects on inducing ferroptosis at various times. But some argued that ferroptosis was not observed in the mouse embryonic fibroblasts treated with sulfasalazine [86]. One reasonable interpretation was the discrepancy of different cell lines on the sensitivity to ferroptosis.

### Artesunate

ART and its derivatives can produce ROS and cause oxidative stress in cancer cells. In pancreatic ductal adenocarcinoma, head and neck cancers (HNCs), and ovarian cancer cells, the mechanism underlying the antitumor effect of ART was ferroptosis-induction [87]. However, because of the activation of Nrf2-antioxidant response element signaling pathway, the ferroptosis induction of artesunate can be partially attenuated in some cisplatin-resistant HNCs [88]. So Nrf2 inhibition via silencing Keap1 helps the reversal of ferroptosis resistance to artesunate in HNC cells.

### Temozolomide

TMZ markedly induces system xc- expression via the activation of a*ctivating transcription factor 4 (*ATF4) and Nrf2 pathway in glioblastoma multiforme (GBM) cells [89]. Cystathionine γ-lyase (CTH), an enzyme in the transsulfuration pathway, is induced after temozolomide treatment, which can supply Cys when system xc- is blocked. Based on the finding of erastin facilitating ferroptotic cell death to temozolomide, thus, the combination of TMZ and erastin maybe a promising therapy in GMB treatment [90].

### Cisplatin

From the screening among five chemotherapeutic drugs, cisplatin was found as a ferroptosis-inducer. Cisplatin exerts its cytotoxic effects on A549 and HCT116 cells to undergo ferroptosis by reduced GSH depletion together with GPXs inactivation [19].

## Targeted agents

### Sorafenib

Sorafenib was first identified as a ferroptosis-inducer in HCC cell lines [91]. System xc- inhibition and GSH depletion, the accomplices for ROS accumulation, were the main mechanism for sorafenib-induced ferroptosis. Haloperidol, as a sigma receptor 1 antagonist, can bolster erastin and sorafenib-induced ferroptosis by stimulating cellular iron accumulation, GSH depletion, and lipid peroxidation [92]. But the overactive p62-keap1-Nrf2 pathway will weaken the ferroptosis process, owing to the target genes of Nrf2 including heme oxygenase-1 (HO-1), FTH1, and quinone oxidoreductase-1 (NQO1) which can directly inhibit ROS accretion. Nrf2 inhibition using genetic tools or drugs could remarkably reinforce the anti-tumor effect of sorafenib [93].

### Lapatinib and BAY87–2243

Lapatinib is a tyrosine kinase inhibitor. It can incite ferroptosis in breast cancer cells when it was used together with siramesine [58]. BAY87-2243, a robust inhibitor of NADH-coenzyme Q oxidoreductase, can promote ferroptosis in a dose-dependent manner on a series of BRaf (V600E) melanoma cell lines [94].

## Others

### Lanperisone

Lanperisone promotes ROS production to kill K-Ras-mutant mouse embryonic fibroblasts in ferroptotic ways. And it also induces lung cancer cell ferroptotic death by inhibiting Cys2 uptake in the mouse model [95].

Besides the clinically approved drugs, two antibiotics such as salinomycin and ironomycin can promote ferroptosis in colon cancer cells via interfering with iron metabolism allowing for ROS production [65]. Other natural compounds such as bromelain [50], baicalein, artenimol, artemisinin, cotylenin A (CN-A) [11], and various vitamins can regulate cell ferroptotic death by acting on the lipid peroxidation and ROS occurrence (Table 3). Numerous nanomaterials have also been prospered for ferroptosis-based cancer therapy. Most of them are iron-based nanomaterials, which can be used as carriers of certain genes, such as P53 and ACSL4, to inhibit or promote the expression of certain critical molecules correlated with ferroptosis [55, 94, 96].

**Table 3:** Drugs and compounds associated with ferroptosis

Category	Drugs/Compounds	Target	Mechanism	Application	Cell lines	Effect	Ref
Chemothe-rapeutic agents	Sulfasalazine	System xc-	Inhibit Cys2 uptake via system xc-	SASP enhanced ferroptosis induced by piperlongumine02 (PL)	BJeLR/HT1080	Induce	[8]
					HT1080/Calu-1		[108]
					HCT116, CX-1, PANC1		[22]
	Artesunate	Fe	React with excess intracellular iron to promote the production of ROS	The cisplatin-resistant cancer cells were less sensitive to artesunate-induced ferroptosis	HN3, HN4, HN9	Induce	[88]
					Panc-1, COLO357		[109]
					BxPC-3, AsPC-1		[110]
	TMZ	System xc-	TMZ induces xCT expression via Nrf2 and ATF4 activation pathway	The efficacy of TMZ can be potentiated after combination with erastin and SASP. SASP potentiates chemo-sensitivity of TMZ in xCT knockdown gliomas	F98, U251	Inhibit	[90]
					u87-MG, GBM-n6, GBM-n15, a172, T98G		[89]
	Cisplatin	GSH-GPXs	The depletion of reduced GSH and inactivation of GPXs	Erastin enhances the effect of cisplatin in NSCLCs.	A549, HCT116	Induce	[19]
					A2780		[111]
Targeted agents	Sorafenib	System xc-	Inhibit system xc--mediated Cys2 import, leading to glutathione depletion and the iron-dependent accumulation of lipid ROS	DFX remarkably reduced the toxicity of sorafenib in an HCC cell line	HT-1080	Induce	[65]
					Huh7		[71]
					ACHN, PLC/PRF5		[92]
					HSC-LX2, HSC		[60]
	Lapatinib	Fe	Cause ferroptosis through iron transport disruption leading to increased ROS	Knockdown of FPN increased ferroptosis after siramesine and lapatinib treatment	SKBR3, MCF-7, MDA-MB-231	Induce	[58]
					MCF-7, ZR-75-1		[18]
Others	Lanperisone	System xc-	Lanperisone-mediated induction of intracellular reactive oxygen species	The remarkable mechanistic similarities of LP as well as erastin underscore the potential of ROS-mediated therapies as a novel strategy to treat K-ras mutant tumors	K-ras-expressing MEFs	Induce	[95]
	Artenimol artemisinin	_	Increase TFRC gene expression and ROS accumulation	Ferrostatin-1 and the iron chelator deferoxamine led to a significantly reduced cytotoxicity of artenimol	CCRF-CEM	Induce	[11]
	Salinomycin Ironomycin	Fe	Interacts with the iron	Against CSCs derived from breast human mammary epithelial cells	CSCs	Induce	[65]
	Bromelain	ACSL-4	Effectively causes ferroptotic cell death by modulating ACSL-4 levels.	Increased erastin-induced ferroptosis in Kras mutant CRC cells	CT-116, DLD-1	Induce	[50]
	Baicalein	LOX	Suppress both lipid peroxidation and iron accumulation; Selectively activate the Keap1-Nrf2 pathway and inhibit 12/15-LOX	–	PANC1, BxPc3	Inhibit	[11]
	Cotylenin A (CN-A)	_	The combined treatment with CN-A and PEITC synergistically increased ROS levels	CN-A plus PEITC inhibited the proliferation of gemcitabine-resistant PANC-1 cells	MIAPaCa-2, PANC-1	Induce	[11]
	Vitamin E	LOX	Inhibits 15-lipox-ygenase via reduction of the enzyme’s non-heme iron from its active Fe3+ state to an inactive Fe2+ state	–	STHdhQ7/Q7	Inhibit	[112]
	ATRA	LSH	Promotes ferroptosis through decreasing the expression of LSH	–	A549	Induce	[113]
	Vitamin C	miR-93	Significantly increased Nrf2 mRNA and protein expression by decreasing miR-93	Prevents estrogen-induced breast tumor development	MCF-10A, T47D	Inhibit	[38]

## Challenges

How does ferroptosis interact with other cell death at the molecular level and how could these pathways be mapped and integrated into the cellular events?

How does ncRNA regulate the process of ferroptotic cell death?

Can ferroptosis enhance the cell immunogenicity to the host and thereby evoke an adaptive immune response, as shown in necroptotic cell death?

Are the ferroptosis inducers effective in killing the cancer cells in pre-clinical or clinical trials?

## Conclusion & perspective

**Fig. 5:**
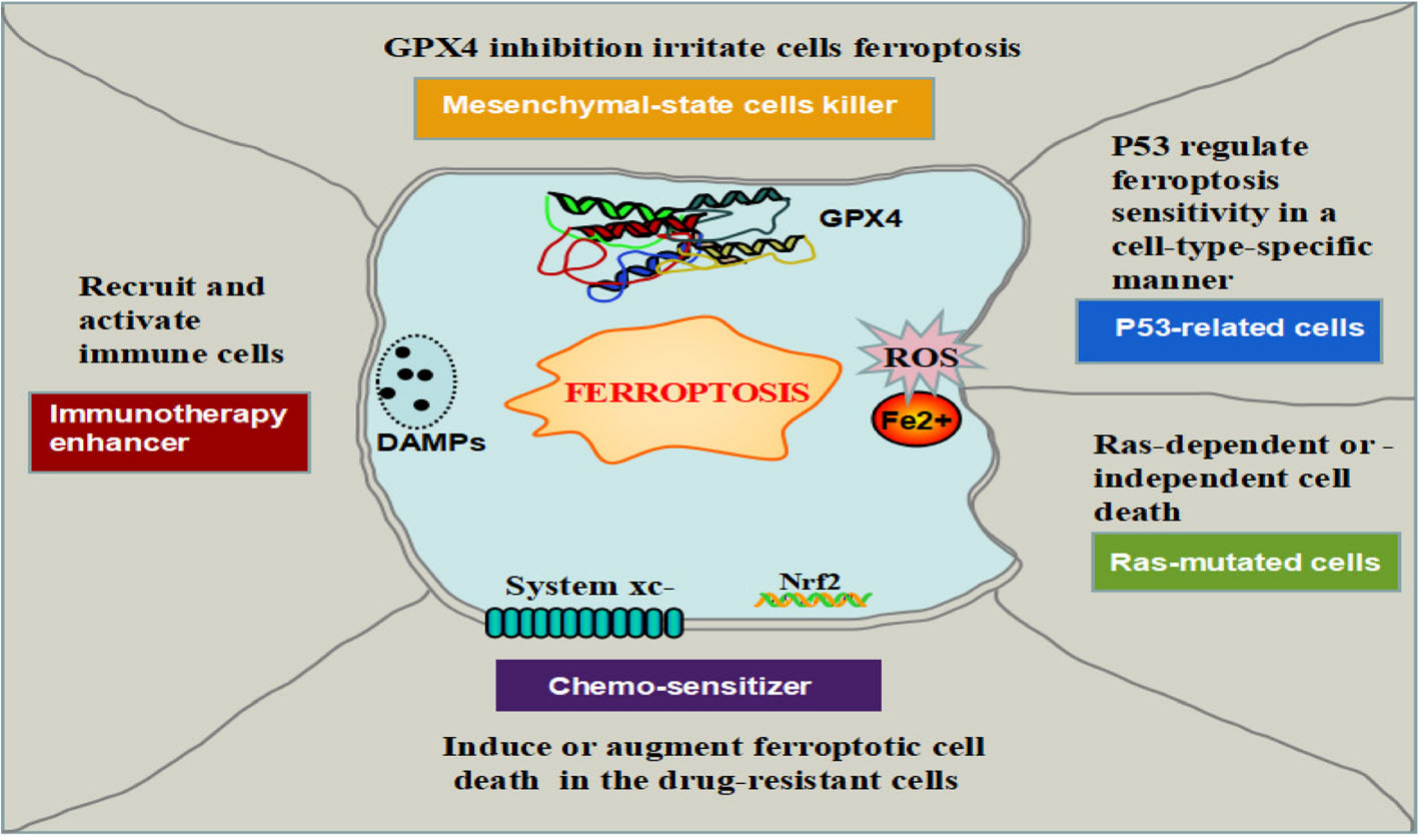
Perspectives of ferroptosis in cancer therapeutics. Ferroptosis can be as a novel cell death for killing mesenchymal-state cells or as an important synergist for immunotherapy and chemotherapy. P53 regulate ferroptosis sensitivity in a cell-type-special way. A large number of small molecules and drugs regulate ferroptosis in a Ras-dependent or -independent manner

Collectively, ferroptosis has taken a full expectation from us to provide a new approach in anti-tumor therapies. Current researches have mainly focused on the eradication of residual or resistant cancer cells, where ferroptotic cell death emerges to be a new cell death for this purpose (Fig. 5). Conspicuously, obtaining a mesenchymal cell state (e.g., epithelial-mesenchymal transition (EMT) or cancer stem cells) has been suggested to determine metastatic dissemination and chemo-resistance [97]. More recently, the cancer cells with the high-mesenchymal state have arisen as a vital mechanism of both acquired and de novo resistance to targeted therapies [98, 99]. This therapy-resistant mesenchymal cancer cells have bred a state of non-oncogene addition to GPX4, which inhibition will intuitively result in ferroptosis. Consistently, persistent cancer cells which are nominated to escape from conventional cytotoxic treatment via a dormant state tumor showed an identically selective dependency on the GPX4 pathway [100, 101]. Therefore, ferroptosis might be considered a viable therapeutic strategy to reverse therapy-resistance in cancer strategy.

Ferroptosis is a kind of programmed necrosis, which is accepted to be more immunogenic than apoptosis. Due to *damage associated* with molecular patterns (DAMPs) (e.g., HMGB1) release, ferroptosis was considered as a pro-inflammatory process [102]. By deliverying chemoattractant signals, ferroptosis hold the ability to recruit and activate immune cells at tumor sites, which provide the possibility of ferroptosis inducer as a suitable enhancer for anti-tumor immunotherapy treatment such as checkpoint-inhibitor [103]. Indeed, a large number of immune cells were observed inside the tumor mass when the mouse tumor xenografts underwent cell ferroptotic death induced by ultrasmall nanoparticles [104]. However, scientist skeptics argued that ferroptosis and necroinflammation did not have an unequivocal relationship [102].

Several studies of drug re-position suggest that “conventional” agents (i.e., SASP and artesunate) have antitumor therapeutic effects by activating ferroptosis [105]. As a chemo-sensitizer by ferroptosis-induction, erastin can be used with various drugs such as cisplatin, temozolomide, doxorubicin/adriamycin, and cytarabine/ara-C in different type cancers. Albeit there is limited knowledge of the elaborated mechanism in the ferroptosis pathway that is engaged by current ferroptosis inducers, ferroptosis may provide a new form of cell death for approaching the reversal of drug-resistance and boosting the host immune system. Although it was promising from the advantages of ferroptosis in cancer therapeutics, ferroptosis is still waiting for formal addressing in a pre-clinical setting and clinical achievability, partially due to the complexity of it observed in different contexts such as P53 or Ras-mutant cancer cells. Another challenge is that ferroptosis induction such as GPX4 inhibitor affects the development and function of nervous system and kidney, by causing GPX4 gene which is fundamental for embryonic development and some adult tissue homeostasis in mice [106]. In addition, another noticeable issue is that the occurrence of ferroptotic resistance, which was originally observed in the Hela cells with the erastin treatment. The resistance mechanism was the HSP27 overactivation by suppressing cytoskeleton-mediated iron absorption [107].

## Abbreviations

ACSL4: Acyl-CoA synthetase long-chain family member 4
AML: Acute myeloid leukemia
AMPK: Adenosine 5′-monophosphate-activated protein kinase
ART: Artesunate
As-SLC7A11: Antisense lncRNAs
ATF4: A*ctivating transcription factor 4*
Atg5: Autophagy-related 5
BAP1: Breast cancer susceptibility gene 1-associated protein 1
BECN1: Beclin1
BID: BH3 interacting domain death agonist
*CARS*: *Cysteinyl-tRNA synthetase*
CD44v: CD44 variant
CDO1: Cysteine dioxygenase type 1
CHOP: C/EBP-homologous protein
CISD1: CDGSH iron sulfur domain 1
c-Myb: C*ellular homolog*
C-Myc: Cellular myelocytomatosis oncogene
CN-A: Cotylenin A
CoQ10: Coenzyme Q10
CTH: Cystathionine γ-lyase
Cul3: Cullin-3
Cys: Cysteine
Cys2: Cystine
DAMPs: D*amage*-*associated* molecular patterns
DDP4: Dipeptidyl-peptidase-4
DHA: Docosahexaenoic acid
DLBCL: Diffuse large B-cell lymphoma
DMT1: Divalent metal transporter 1
EGLN1: Egl nine homolog 1
ELAVL1: Embryonic lethal, abnormal vision, Drosophila-like 1
EMT: Epithelial-mesenchymal transition
ERK: E*xtracellular signal-regulated kinase*
FADS2: Fatty acid desaturase 2
FIN: Ferroptosis *inducing agents*
Flt3: Fms-like tyrosine *kinase* 3
FPN: Ferroportin
FTH1: F*erritin heavy chain* 1
FTL: F*erritin light* chain
G3BP1: Ras GTPase-activating protein-binding protein 1
GBM: Glioblastoma multiforme
Gln: Glutamine
GLS2: Glutaminase 2
Glu: Glutamate
GLUT1: Glucose transporter 1
GOT1: Glutamic-oxaloacetic transaminase 1
GPX4: Glutathione peroxidase 4
GSC: Glioblastoma stem cells
GSH: Glutathione
HIF: Hypoxia-inducible transcriptional factor
HNCs: Head and neck cancers
HO-1: Heme oxygenase-1
HSF1: Heat shock factor-1
HSPB1: Heat shock protein beta-1
IPP: Isopentenyl pyrophosphate
IRE: Iron-responsive element
IREB2: Iron response element binding protein 2
*Keap1*: *Keleh-like ECH-associated protein 1*
LDL: Low-density lipoprotein
LncRNA: Long non-coding RNA
LPCAT3: Lysophosphatidylcholine acyltransferase 3
LSH: Lymphoid-specific helicase
MEK: Ras-mitogen-activated protein kinase kinase
MON-P53: Metal organic network-P53
MT-1G: Metallothionein-1 g
MUC1-C: Mucin 1 C-terminal
MVA: Mevalonate
NCOA4: Nuclear receptor coactivator 4
NFS1: Cysteine desulfurase
NOX: Nicotinamide adenine dinucleotide phosphate-*oxidase*
NQO1: Quinone oxidoreductase-1
Nrf2: Nuclear factor erythroid 2-related factor 2
*PHKG2*: Phosphorylase kinase gamma 2
PIK3C3: Phosphatidylinositol 3-kinase catalytic subunit type 3
*PL-*PUFAs: *P*hospholipid-polyunsaturated fatty acids
*PtdIns3K*: *Phosphatidylinositol 3-kinase*
PUFAs: Polyunsaturated fatty acids
PUMA: P53 *upregulated modulator of apoptosis*
RB: R*etinoblastoma*
ROS: R*eactive oxygen species*
RRM: *RNA recognition motif*
RSL3: Ras-selective lethal small molecule 3
SAT1: Spermidine/spermine N1-acetyltransferase 1
SCD1: Stearoyl-CoA desaturase 1
Se: Selenium
SLC1A5: S*olute* c*arrier* f*amily 1* member 5
SLC3A2: Solute carrier family 3 member 2
SLC7A11: Solute carrier family 7 member 11
SOD1: Superoxide dismutase 1
SQS: Squalene synthase
STEAP3: Six-transmembrane epithelial antigen of the prostate 3
TF: Transferrin
TFR1: Transferrin receptor 1
TICs: Tumor-initiating cells
TP53: T*umor suppressor P53*
TRAIL: Tumor necrosis factor-related apoptosis-inducing ligand
UPR: Unfolded protein response
VDAC2/3: Voltage-dependent anion channel 2/3
xCT: S*ystem xc*-
α-KG: α-ketoglutarate
